# The Effects of a School-Based Physical Activity Program on Physical Fitness in Egyptian Children: A Pilot Study from the DELICIOUS Project

**DOI:** 10.3390/children11070842

**Published:** 2024-07-10

**Authors:** Osama Abdelkarim, Noha El-Gyar, Amira M. Shalaby, Mohamed Aly

**Affiliations:** 1 Faculty of Physical Education, Assiut University, Assiut 71515, Egypt; 2 Department of Pediatric, Faculty of Medicine, Assiut University, Assiut 71515, Egypt

**Keywords:** health promotion, physical activity, physical fitness, DELICIOUS project, Egyptian children, pilot study

## Abstract

Background: Ensuring the physical fitness of Egyptian children is of paramount importance to their overall well-being, given the unique socio-cultural and educational barriers they face that may hinder their active participation. As part of the DELICIOUS project, the “Be Fit Program” aims to increase the level of physical fitness among Egyptian school-aged children. This study explores the effectiveness of a structured, six-week physical activity (PA) program in improving various facets of physical fitness in children, including body composition, speed, coordination, muscular strength, and cardiovascular endurance. With the increasing prevalence of sedentary lifestyles, such efforts are imperative to improve overall health outcomes. Methods: A cohort of 125 children, aged 8.50 to 12.25 y (mean age 10.19 ± 1.03 y), participated in the study. Their body composition, speed, coordination, strength, and aerobic fitness were assessed before and after the Be Fit Program using the revised International Physical Performance Test Profile. Paired *t*-tests were used to detect changes between the pre- and post-tests. Results: Following the six-week intervention, statistical analyses revealed significant improvements in coordination and lower body strength (*p* < 0.01). Aerobic endurance showed marginal improvements, approaching statistical significance (*p* = 0.06). Conversely, there were no statistically significant changes in body composition, speed, or upper body strength (*p* > 0.05). Conclusions: The study confirms that tailored, non-competitive physical activities can positively influence specific fitness components in Egyptian children. However, achieving holistic improvements across all targeted fitness domains may require further strategic adjustments or a longer program duration. This pilot study underscores the importance of culturally tailored, school-based PA programs and highlights the continued need for research and program refinement to comprehensively improve children’s fitness in the Egyptian context.

## 1. Introduction

The physical fitness in children is increasingly recognized as a cornerstone of public health, with profound implications for long-term well-being and development [1,2]. Physical activity (PA) in childhood not only provides immediate health benefits but also sets the stage for a healthier lifestyle in adulthood [3,4]. From a physical perspective, regular PA improves cardiovascular health [1], enhances muscle and bone strength [5], betters body composition [6], and elevates overall fitness levels [7]. Socially, participation in PA promotes teamwork [8], boosts social skills [9], and strengthens peer relationships [10]. Psychologically, it is associated with improvements in mood, a reduction in symptoms of depression and anxiety, increased self-esteem, and better cognitive function [11]. The diverse benefits highlight the critical importance of integrating regular PA into the daily routines of children. However, there is a worrying global trend of declining PA and fitness levels among children worldwide, which mirrors concerns in Egypt [12,13]. In Egypt, where unique cultural and socioeconomic dynamics intersect, the importance of childhood physical fitness goes beyond mere health outcomes. Within this distinct socio-cultural and educational milieu, Egyptian children face specific barriers to participation in PA, including limited access to recreational facilities, socio-economic constraints, and educational structures. Specifically, socio-economic constraints can limit access to necessary sports equipment and facilities, further hindering regular participation in PA. Additionally, the theoretical background presented by Sáez et al. (2022) in their study on the health benefits and participation barriers of horseback riding can provide insights into similar barriers in different contexts. They identified key barriers such as lack of facilities and socio-economic challenges, which can be extrapolated to the Egyptian context [14]. In addition, the education system in developing countries, which prioritizes academic achievement, often overshadows the importance of physical education and extracurricular activities [15,16,17]. This emphasis on academic achievement at the expense of physical well-being may encourage sedentary lifestyles and suboptimal fitness levels among Egyptian children. As a result, school-based PA programs emerge as critical, providing a structured and accessible way to promote multiple dimensions of physical fitness. When integrated into the educational curriculum, these programs provide a comprehensive approach to physical fitness, which positively impacts academic performance and socio-relational competence. Incorporating such activities into daily routines enables holistic development, enhancing collaboration, boosting self-esteem, and promoting positive peer interactions. Consequently, these benefits may lead to improved academic engagement and the development of interpersonal skills that transcend the benefits of physical health alone. The significance of this study lies in its potential to provide crucial evidence on the effectiveness of tailored, school-based PA interventions in improving children’s physical fitness.

This study is part of the DELICIOUS project, which seeks to promote healthier lifestyles among children and adolescents in the Mediterranean region, focusing on improvements in both dietary habits and PA [18,19,20]. At the heart of this initiative is the Be Fit Program, which has been carefully designed to target key fitness parameters such as motor endurance, strength, speed, and coordination, while taking into account individual abilities and preferences. Inspired by the Build Our Kids’ Success (BOKS) Elementary PA Plans, the program prioritizes the use of activities and resources that promote adherence to healthier lifestyles [21]. The Be Fit Program is a pilot intervention that specifically aims to identify collaborative strategies between universities and communities to implement innovative and effective health promotion initiatives in primary schools. This endeavor is of particular importance in developing countries such as Egypt, where there is an apparent lack of effective, evidence-based interventions to improve physical fitness in children. While previous studies have predominantly focused on the quantitative facets of PA, they often overlook qualitative elements such as enjoyment, individualization, and non-competitive exercises, all of which profoundly influence a child’s motivation and sustained engagement [22,23]. By adopting a holistic approach that encompasses both the physical and psychological dimensions of fitness, the Be Fit Program aims to bridge these gaps and promote sustainable healthy lifestyles among children and adolescents throughout the Mediterranean region.

The complexity of physical fitness is underlined by the different rates at which its components adapt in response to exercise. Within a six-week exercise intervention, some facets of fitness, such as aerobic capacity, may improve rapidly due to physiological changes such as increased capillary density, enhanced mitochondrial function, and improved enzymatic activity in muscles [24,25]. These changes can occur within a few weeks of consistent aerobic training. Similarly, gains in coordination and motor skills, which are crucial for activities such as lateral jumps, are typically more immediately achievable, particularly in children. This phenomenon has been attributed to the critical window of motor skill acquisition during adolescence, when neural adaptations and motor learning can lead to relatively rapid gains [26,27,28]. Conversely, substantial improvements in muscular strength and endurance often take longer than six weeks, as they depend on both neural adaptations and hypertrophic changes in muscle fibers, processes that are generally more gradual [29,30]. Furthermore, the rate of muscular development varies considerably between individuals, particularly in children who are at different stages of physical maturation and development [31].

This study aims to evaluate the influence of the Be Fit Program—a six-week PA intervention—on body composition (BMI) and physical fitness levels of Egyptian children. Several fitness components were assessed using the revised International Physical Performance Test Profile (IPPTP) 6–18. The primary hypothesis guiding this research is that participation in the Be Fit Program, a tailored and non-competitive PA intervention, will lead to notable improvements in various components of physical fitness in children, with certain aspects showing more pronounced improvement than others. This hypothesis is based on the premise that tailored, engaging physical activities can have a greater impact on specific fitness domains. The methodological framework, which includes pre- and post-intervention measurements, allows for a comprehensive analysis of the program’s influence on different facets of children’s physical fitness and body mass index (BMI). This study promises to provide invaluable insights for public health policy, and to serve as a blueprint for future interventions aimed at increasing physical fitness among the pediatric population, thus charting a course towards a healthier future for Egyptian children.

## 2. Materials and Methods

### 2.1. Participants

A total of 137 children were initially recruited from local schools in Assiut governorate, Egypt. One hundred twenty-five participants (mean age = 10.19 y, SD = 1.03) completed all required measures. Inclusion criteria required the enrolment of children between the ages of 9 to 11 y who had no health problems that could hinder or jeopardize their participation in a PA program. In addition, participants with acute or chronic medical conditions that limit PA were excluded to ensure the safety and suitability of the Be Fit Program for all involved. To ensure a balanced representation, both boys and girls were included to reflect the gender diversity of the school population. Prior to commencement, informed consent was obtained from each child’s parents or guardian in accordance with strict ethical protocols. All participants and their guardians properly duly completed and signed informed assent/consent forms in accordance with the Institutional Review Board of Assiut University. In particular, the children selected were typically developing, with no reported history of chronic disease or physical disability that might affect their ability to participate in the prescribed physical activities outlined in the Be Fit Program.

Analyses were conducted on a refined sample of 125 participants after excluding individuals who (1) lacked data for both pre-and post-measures (*n* = 8) and (2) attended less than 75% of the PA program sessions (*n* = 4). Prior to data analysis, a comprehensive sample size calculation was performed using G*Power 3.1.9.7 [32]. This calculation determined that a minimum of 122 participants would provide sufficient statistical power to detect small to moderate effect sizes (*d* = 0.3) [33], assuming a desired statistical power = 0.95 and alpha = 0.05. Therefore, our final sample size of 125 was considered adequate to achieve the required power. The demographic characteristics of the final sample are shown in Table 1.

### 2.2. Be Fit Program

The Be Fit Program, derived from the BOKS Elementary PA Plans [21], was a carefully structured six-week PA program. This initiative included a comprehensive range of activity plans tailored to improve different functional movement skills. Each session consisted of a carefully designed sequence, including warm-up routines, skill introductions, movement-related activities and engaging games designed to reinforce the skills introduced. Sessions were held three times a week, with each session lasting between 40 and 45 min. To ensure seamless delivery and supervision, at least one researcher was present during all sessions. Two senior undergraduate students specializing in physical education carefully curated the activities and actively engaged with the children, providing constant encouragement and motivation. The program’s diverse repertoire of exercises aimed to improve motor endurance, strength, speed, and coordination, and included activities such as running, jumping, and various strength-building exercises (for more detailed information on the BOKS Elementary PA Plans, see https://trainerhub.activekids.org/s/, accessed on 25 May 2024). Central to the Be Fit Program was its tailored approach. Initial assessments of each child’s baseline fitness level facilitated the tailoring of activities to their individual fitness abilities and skills. This individualized approach ensured that the exercises provided an appropriate level of challenge while remaining achievable, thereby encouraging sustained engagement and a sense of achievement among participants. In addition, the Be Fit Program supported the non-competitive ethos inherent in the BOKS model. The program prioritized the enjoyment of PA over peer competition, with a deliberate emphasis on individual progress and goals. This philosophy was underpinned by a culture of celebrating personal achievement, with trained coaches, including postgraduate students from the Faculty of Physical Education, playing a key role. By recognizing and celebrating individual milestones, the program cultivated a supportive and nurturing environment in which children were encouraged to participate enthusiastically, regardless of their initial fitness levels.

### 2.3. Physical Fitness

IPPTP is a robust and validated instrument tailored to the assessment of physical fitness, carefully designed for practical use [34]. Based on the methodologies of Bös and Mechling [35] and the German Motor Test 6-18 [36], this tool comprises eight test items that comprehensively cover the five fundamental dimensions of physical fitness: endurance, strength, speed, coordination, and flexibility. In our study, we used six fitness tests that were carefully designed to assess different aspects of physical fitness. The 20 m dash tests speed by measuring the time (in milliseconds) taken to sprint 20 m using a stopwatch. The sideways jumping test assesses agility and coordination by recording the number of sideways jumps completed within 15 s. Upper body strength and endurance are assessed by the push-up test, which records the total number of push-ups performed in 40 s. Similarly, the sit-ups test measures core strength and endurance by counting the number of sit-ups performed at the same time. The standing long jump test assesses lower body strength by measuring the distance jumped from a standing position in centimeters. Finally, the 6 min run test measures cardiovascular endurance by recording the total distance covered in six minutes, measured in meters. Each test is conducted under standardized conditions to ensure consistency and reliability in assessing the main dimensions of physical fitness, which include endurance, strength, speed, coordination, and flexibility. In addition to these test items, key constitutional data such as height, weight, and BMI were carefully recorded using a FullMedi scale (Full Medical Co., Ltd., Hefei, China). Table 1 provides a brief overview of the test items used in our study. For further explanation of these test items, the reader is referred to the available manuals [34,36].

### 2.4. Procedure

Informed consent was carefully obtained from the parents and children before the study began. Potential participants were thoroughly informed of the aims and procedures of the research prior to enrolment in the Be Fit Program. Each participant received a comprehensive information packet delineating the research protocols, inclusive of parental consent and child assent forms. The program was conducted after school hours on school premises and was supervised by two experienced postgraduate physical education students. At the start of the program, baseline data were carefully collected, including age, height, and weight, BMI was calculated (body weight [kg]/body height [m]^2^). Both before and after the Be Fit Program, participants underwent the IPPTP 6–18, with explicit encouragement to exert maximum effort during these assessments to ensure an accurate assessment of their physical fitness gains.

### 2.5. Statistical Analysis

Summary statistics were used to report fitness and body composition. Normality of data distribution was confirmed using the Shapiro–Wilk normality test. Mean differences in the fitness tests (including the 20 m dash, standing long jump, jumping sideways, push-ups, sit-ups, and 6 min run) and body composition variables between the pre- and post-intervention were assessed using two-tailed paired *t* tests. The significance level was set at *p* < 0.05. All statistical analyses were performed using SPSS version 25.0 (IBM, Corp., Armonk, NY, USA).

## 3. Results

### 3.1. Participant Demographics

The study cohort consisted of 125 children, 56 of whom were female (44.8%) and ranged in age from 8.50 to 12.25 y, with a mean age of 10.19 ± 1.03 y. The distribution by grade was as follows: Grade 3 (47 children, 37.6%), Grade 4 (37 children, 29.6%), and Grade 5 (41 children, 32.8%). On average, participants were 1.38 m tall (SD = 0.07) and weighed 35.55 kg (SD = 9.88). Full demographic details can be found in Table 2.

### 3.2. Fitness Test Performance

The results of the fitness tests conducted before and after the Be Fit Program are summarized in Table 3. Paired *t*-tests showed no significant changes in BMI, 20 m dash, push-ups, and sit-ups (*p* < 0.05). However, statistically significant improvements were observed in jumping sideways, with scores increasing from 21.34 ± 3.36 to 21.71 ± 3.23 counts (*p* = 0.02), and in the standing long jump, with distances increasing from 107.42 ± 13.46 to 108.70 ± 12.85 cm (*p* = 0.03). There was also a marginally significant improvement in the 6 min run test, with the distance covered increasing from 943.88 ± 195.19 to 959.73 ± 234.02 m (*p* = 0.06).

## 4. Discussion

This pilot study aimed to evaluate the effectiveness of the Be Fit Program, an individualized school-based PA initiative, in improving the physical fitness of Egyptian children. Our results show notable improvements in specific fitness components, particularly coordination (as evidenced by improved performance in the lateral jump test), and lower body strength (as evidenced by improvements in the standing long jump). However, the program did not have significant effects on other fitness parameters such as speed, upper body strength, and overall body composition (as indicated by BMI). These results suggest that while the program was effective in targeting certain facets of physical fitness, further interventions of longer duration or greater intensity may be required to achieve substantial improvements in other areas.

Our results confirmed our initial expectations, showing varying degrees of responsiveness between different fitness components following a 6-week exercise intervention. In particular, we observed no significant changes in the dash, push-up, and sit-up tests. However, there were notable improvements in aerobic fitness and statistically significant improvements in both the side jump and 6 min run tests. The data from our study, which highlight the differential response of different fitness components to the 6-week PA intervention, provide valuable insights into the dynamics of physical fitness development in Egyptian children. The significant improvements observed in coordination and lower body strength are likely due to the Be Fit Program’s emphasis on functional movement and motor skill development. These findings are consistent with previous research suggesting that motor skill-related fitness components can be effectively improved through targeted PA in a relatively short period of time, due to the rapid adaptability of the neuromuscular system in children [37]. Given the ongoing neurological maturation of children and the plasticity of their developing neuromuscular systems [38], activities that focus on coordination and agility can rapidly improve motor skills, balance, and coordination, crucial facets of childhood development [39]. The increased performance in both the side jump and standing long jump tests underscores the success of the intervention in these specific areas, highlighting the responsiveness of neuromuscular coordination and agility to the Be Fit Program. These improvements potentially contribute to improved overall motor performance and physical well-being. The observed improvements in coordination and explosive strength underline the effectiveness of the Be Fit Program over 6 weeks in promoting these specific areas of physical fitness. This may be due to the program’s targeted activities to improve motor skills such as jumping and dynamic movements, which play a key role in children’s physical development.

The slight nonsignificant enhancement observed in aerobic endurance, as assessed by the 6 min run test, suggests that a six-week period may not be sufficient to induce substantial changes in aerobic capacity, a parameter that typically requires sustained and progressive overload to improve [40,41]. However, this marginal improvement may also indicate the relatively rapid adaptability of children’s cardiovascular and respiratory systems to aerobic exercise. Aerobic fitness, which is characterized by the body’s efficiency in using oxygen, can improve through the increased heart and lung capacity and increased blood flow, even within a relatively short intervention period such as six weeks [42]. This is particularly true in children, whose bodies are more adaptable and respond more quickly to aerobic stimuli [24,43,44,45]. Although the improvement in aerobic endurance did not reach statistical significance, this observation highlights the need for either a longer intervention period or an increased aerobic component within the program to achieve more pronounced aerobic improvements.

The 6-week PA intervention did not result in changes in body composition, consistent with observations that such interventions often have minimal impact on reducing childhood obesity [46]. Similarly, the association between physical fitness and BMI in children has been inconsistent [47]. Conversely, sustained improvements in cardiovascular fitness and BMI have been reported following long-term PA interventions [48,49,50,51,52]. In addition, one study documented a reduction in BMI following a 14-week intervention combining dietary, behavioral, and PA components [53]. The lack of observed changes in BMI in our study may be due to baseline weight status, as PA interventions tend to be more effective in reducing BMI in obese children compared to normal weight or overweight children [52]. In addition, factors such as age, gender, and pre-intervention PA levels have been identified as important determinants of intervention effectiveness [54]. Younger children, in particular, have a greater degree of metabolic flexibility, leading to more pronounced and rapid changes in response to PA. This phenomenon is particularly pronounced in prepubertal children, whose bodies and metabolisms adapt to exercise more efficiently than those who are in or have completed puberty [55]. In girls, the onset of puberty, which occurs earlier than in boys, leads to hormonal changes that affect fat distribution, muscle growth, and metabolism. In particular, significant improvements in BMI with PA have been documented in prepubertal girls due to their increased responsiveness to exercise before the onset of major hormonal changes [56,57]. In conclusion, the effectiveness of PA interventions in modifying body composition, particularly BMI, is influenced by a complex interplay of factors, including intervention duration, participants’ baseline weight status, and individual characteristics such as age, sex, and initial PA levels. Long-term, comprehensive interventions that take these variables into account hold promise for improving child health and combating obesity.

The lack of significant changes in speed and upper body strength within the Be Fit Program highlights the need for longer intervention periods and targeted training modalities. These fitness components rely on gradual physiological adaptations, such as muscle hypertrophy and metabolic improvements, which require longer durations and specific training stimuli to manifest noticeable improvements. In essence, the duration and intensity of the program may have been insufficient to induce changes in these specific areas of fitness. This observation is consistent with existing literature suggesting that short-term interventions may not adequately address certain fitness parameters in children [58]. The development of muscular strength and endurance typically requires progressive overload and sustained training over time [59,60], particularly in pediatric populations where muscular growth is influenced by growth and maturation stages [61]. Children’s muscles respond differently to strength training compared to adults [62], so significant gains in strength and endurance may require durations longer than six weeks, especially without specific emphasis on these areas.

In addition, unique cultural and environmental factors prevalent among Egyptian children, such as high academic pressure and limited opportunities for PA outside of school, are likely to have influenced the results of the Be Fit Program. In Egypt, the emphasis on academic achievement often takes precedence over physical education, which may encourage sedentary lifestyles among children. Additionally, the lack of accessible and safe recreational spaces limits the range of physical activities available, restricting opportunities to engage in the variety of exercises essential for the development of different fitness components [63,64]. To effectively address these challenges, interventions such as the Be Fit Program need to include extending the duration and intensity of sessions, incorporating targeted exercises to address key fitness components, and better integrating PA into community spaces and school curricula to alleviate academic constraints. For instance, introducing after-school sports programs or weekend community fitness events can provide additional PA opportunities. Collaborating with local schools to integrate short, frequent exercise breaks during the school day can help balance academic and physical activity demands. Furthermore, engaging community leaders and parents to raise awareness of the benefits of physical fitness and advocating for policy changes that prioritize physical education in schools are essential steps [65]. Building partnerships with local community centers or sports clubs can also enhance the accessibility and appeal of PA for children. By adopting this strategy, such programs can be more effective in promoting children’s sustainable physical development and fitness, ultimately cultivating a healthier and more active generation.

In summary, this pilot study evaluated the effectiveness of the “Be Fit Program”, a school-based PA initiative aimed at improving the fitness levels of Egyptian children. The study found significant improvements in coordination and lower body strength, as evidenced by improved performance in the jumping sideways and standing long jump tests. However, other fitness components such as speed, upper body strength, and BMI did not show significant changes. While the program marginally improved aerobic endurance, it did not produce significant improvements in aerobic capacity, suggesting a potential need for a longer or more intensive intervention period. Cultural and environmental factors unique to Egyptian children, including high academic pressure and limited opportunities for PA, may have influenced these results. The lack of change in speed, upper body, and core strength suggests that short-term interventions may not be sufficiently influential in these areas. This highlights the importance of tailored interventions to effectively target specific fitness components in children. However, it is important to recognize the limitations of the study, particularly the lack of a control group. Therefore, the results should be interpreted with caution and future studies should consider including a control group for a more robust analysis. Despite its limitations, this pilot study highlights the potential of school-based programs such as the Be Fit Program to promote meaningful improvements in children’s physical fitness within a limited timeframe. To achieve more comprehensive fitness improvements, future iterations of the program could integrate a wider range of activities focusing on upper body and core strength, while also implementing strategies to encourage greater and sustained participation in PA outside of structured sessions. Further research should explore the longitudinal effects of continued participation in such programs, possibly incorporating modifications such as longer duration or more frequent sessions per week. Moreover, qualitative assessments of participants’ motivation, enjoyment, and overall engagement with the program could provide deeper insights into how these factors influence the effectiveness of school-based PA initiatives. Additionally, future studies should comprehensively consider other critical elements like sleep and nutrition to better understand their combined impact on fostering a healthy future for children and adolescents. Lastly, although socioeconomic background could potentially influence participation in PA, our study did not include a measure of socioeconomic status. However, given that all participating children were enrolled in a national private school and predominantly belonged to the middle class, it was difficult to analyze its impact. Nevertheless, future research is encouraged to incorporate considerations of socioeconomic background when examining the effects of PA programs in developing countries, as it may provide valuable insights into how these factors influence program outcomes.

## 5. Conclusions

In conclusion, the DELICIOUS pilot study on the implementation of the Be Fit Program sheds light on the potential effectiveness of school-based PA interventions in improving specific dimensions of physical fitness in children. The program resulted in significant improvements in coordination and strength, accompanied by marginal improvements in aerobic endurance among Egyptian children. However, the lack of significant changes in speed of action, strength endurance, and body composition suggests the need for program adaptations or longer intervention periods to achieve comprehensive fitness improvements. Furthermore, the limited impact on specific fitness components may reflect broader cultural and environmental factors that influence children’s overall PA levels and health, including sedentary lifestyles, academic pressures, and limited access to recreational facilities. This study highlights the importance of tailored, non-competitive PA in school settings and provides a basis for future research and program development aimed at promoting holistic physical fitness in children. The findings provide a valuable contribution to public health initiatives targeting children’s health and well-being and advocate the introduction of personalized PA programs in educational settings. By emphasizing individualized approaches to physical fitness, such initiatives can better address the diverse needs and abilities of children, ultimately promoting a healthier and more active generation.

## Figures and Tables

**Table 1:** Test items of the IPPTP-R.

Dimension	Test Item	Unit
Speed	20 m dash	Second
Coordination	Jumping sideways	Number of jumps in 15 s
Strength	Push-ups	Number in 40 s
	Sit-ups	Number in 40 s
	Standing long jump	Centimeter
Endurance	6 min run	Meter

**Table 2:** Participant demographic variables.

Measure	All (*n* = 125)	Female (*n* = 56)	Male (*n* = 69)
Age (y)	10.19 (1.03)	10.12 (1.01)	10.24 (1.04)
Grade			
Grade 3 (%)	37.6	41.1	34.8
Grade 4 (%)	29.6	28.6	30.4
Grade 5 (%)	32.8	30.4	34.8
Height (meter)	1.38 (0.07)	1.36 (0.08)	1.38 (0.75)
Weight (kg)	35.55 (0.98)	34.45 (9.28)	36.45 (10.32)
Underweight (<5th)	4.8%	1.8	7.2
Normal BMI (5th–85th)	63.2%	69.6	58.0
Overweight or obese (≥85th)	15.2%	19.6	11.6
Obese (≥95th)	16.8%	8.9	23.2

**Table 3:** Paired *t*-test analysis of mean differences in fitness tests between pre-and post-intervention assessments, including means (SDs).

Variable	Pre-Program	Post-Program	*t*	*p*
BMI	18.54 (3.65)	18.49 (3.71)	1.59	0.12
20 m dash (s)	4.81 (0.31)	4.78 (0.29)	1.70	0.09
Jumping sideways (n)	21.34 (3.36)	23.11 (3.27)	20.55	<0.01
Push-ups (n)	16.34 (3.10)	16.47 (3.65)	1.12	0.27
Sit-ups (n)	22.52 (3.42)	22.60 (4.61)	0.56	0.58
Standing long jump (cm)	107.42 (13.46)	113.70 (11.16)	23.67	<0.01
6 min run (meter)	943.88 (195.19)	959.73 (234.02)	1.88	0.06

## Data Availability

The raw data supporting the conclusions of this article will be made available by the authors, without undue reservation.
